# NIA Division of Extramural Activities

**DOI:** 10.1093/geroni/igaf122.1555

**Published:** 2025-12-31

**Authors:** Kenneth Santora

**Affiliations:** National Institute on Aging, NIH, Bethesda, Maryland, United States

## Abstract

Dr. Santora will discuss the work of the NIA Division of Extramural Activities. Dr. Santora will also be available for small group discussion.

